# Targeting Bone Remodeling by Isoflavone and 3,3′-Diindolylmethane in the Context of Prostate Cancer Bone Metastasis

**DOI:** 10.1371/journal.pone.0033011

**Published:** 2012-03-07

**Authors:** Yiwei Li, Dejuan Kong, Aamir Ahmad, Bin Bao, Fazlul H. Sarkar

**Affiliations:** 1 Department of Pathology, Barbara Ann Karmanos Cancer Institute, Wayne State University School of Medicine, Detroit, Michigan, United States of America; 2 Department of Oncology, Barbara Ann Karmanos Cancer Institute, Wayne State University School of Medicine, Detroit, Michigan, United States of America; Mayo Clinic College of Medicine, United States of America

## Abstract

Prostate cancer (PCa) bone metastases have long been believed to be osteoblastic because of bone remodeling leading to the formation of new bone. However, recent studies have shown increased osteolytic activity in the beginning stages of PCa bone metastases, suggesting that targeting both osteolytic and osteoblastic mediators would likely inhibit bone remodeling and PCa bone metastasis. In this study, we found that PCa cells could stimulate differentiation of osteoclasts and osteoblasts through the up-regulation of RANKL, RUNX2 and osteopontin, promoting bone remodeling. Interestingly, we found that formulated isoflavone and 3,3′-diindolylmethane (BR-DIM) were able to inhibit the differentiation of osteoclasts and osteoblasts through the inhibition of cell signal transduction in RANKL, osteoblastic, and PCa cell signaling. Moreover, we found that isoflavone and BR-DIM down-regulated the expression of miR-92a, which is known to be associated with RANKL signaling, EMT and cancer progression. By pathway and network analysis, we also observed the regulatory effects of isoflavone and BR-DIM on multiple signaling pathways such as AR/PSA, NKX3-1/Akt/p27, MITF, etc. Therefore, isoflavone and BR-DIM with their multi-targeted effects could be useful for the prevention of PCa progression, especially by attenuating bone metastasis mechanisms.

## Introduction

Prostate cancer (PCa) is a common cancer and the second leading cause of cancer related deaths in men in the United States with an estimated 241,740 new cases and 28,170 deaths are expected in 2012 [1]. The high rate of mortality of PCa is mainly due to the development of metastasis. PCa commonly exhibits its progressive features through the cascades of androgen dependence to castrate resistance with eventual metastasis. Even though the PCa may be considered localized to the prostate, there is still a 15% to 20% incidence of subsequent metastasis [2]. It has been reported that 35% of patients with PCa develop hematogeneous metastases and that bone metastasis of PCa is the most frequent (∼90%) among the hematogeneous metastases [3]. PCa bone metastases have long been believed to be osteoblastic because of the formation of new bone. Therefore, targeting osteoblastic molecules such as endothelin-1, BMP, and Wnt signaling has been considered as strategies for inhibiting PCa bone metastasis [2]. However, recent studies found increased osteolytic activity in the beginning stages of PCa bone metastases [4], [5]. Several growth factors were found to be released from the bone matrix during degradation when PCa cells metastasized to the bone. Moreover, cancer cells could spread to the bone and utilize the local cytokine machinery to stimulate osteoclastogenesis, resulting in bone resorption and cancer cell growth [6]. These findings suggest that bone remodeling including osteolytic and osteoblastic processes occurs during PCa bone metastasis and, in turn, favors the growth of PCa cells in the newly formed bone. Therefore, molecular targeting of both osteolytic and osteoblastic mediators would likely inhibit bone remodeling, which could become a newer therapeutic strategy for the inhibition of PCa bone metastasis.

To inhibit osteolytic process, several strategies have been developed including the use of bisphosphonates and targeting the biological regulators of osteoclastogenesis, such as osteoprotegerin (OPG), receptor activator of nuclear factor-κB (RANK) and receptor activator of nuclear factor-κB ligand (RANKL). The most important cytokine machinery, which is involved in bone remodeling and PCa bone metastasis, is OPG/RANK/RANKL signaling [7], [8]. RANKL is expressed by osteoblasts, and it is necessary and sufficient for osteoclastogenesis [9]. RANKL binds to its receptor RANK which is present at the surface of osteoclast precursors, inducing osteoclast formation and activation [9], [10]. Studies have shown that RAW264.7 cells, one of the osteoclast precursor macrophages, could differentiate to osteoclasts when cultured in the presence of RANKL [10]. The major features of osteoclasts include the abilities to absorb bone, to express tartrate-resistant acid phosphatase (TRAP), and to express proteases including matrix metalloproteinases (MMPs) which favor cancer invasion and metastasis [10], [11]. Importantly, the expression of RANKL has also been found in some cancer cells as well as in activated T-cells [6], [11], [12]. Therefore, RANKL signaling has been believed to be a therapeutic target for the inhibition of bone remodeling and bone metastasis [13].

Moreover, several molecules including endothelin-1, BMP, and Wnt have been believed as the important regulators for osteoblast differentiation and bone formation [2], [14]–[16]. The histological studies have shown that osteoblastic lesions of PCa bone metastasis are characterized by deposition of new bones, which are produced by osteoblasts, unorganized and interlaced between foci of cancer cells. In osteoblasts, endothelin-1, BMP, and the molecules in Wnt signaling are highly expressed. In addition, elevated serum levels of bone-specific alkaline phosphatase, a marker of osteoblast differentiation and proliferation, are often observed in cancer patient with bone metastasis [17], suggesting the importance of these molecules in PCa bone metastasis. Therefore, targeting these osteoblastic molecules could inhibit bone remodeling and PCa bone metastasis.

Recently, natural agents have received much attention in the area of cancer research. Isoflavone genistein mainly found in soybean has shown its ability to inhibit cancer cell growth *in vitro* and *in vivo* without toxicity. We have previously found that isoflavone genistein could inhibit NF-κB and Akt activation in cancer cells [18]. Moreover, we have reported that dietary genistein could inhibit PCa in experimental bone metastasis in a SCID-human model [19] and that genistein could potentiate apoptosis inducing effects of chemotherapeutic agents through down-regulation of NF-κB [20]. 3,3′-diindolylmethane (DIM) is another natural agent and mainly found in the members of the family Cruciferae such as broccoli. We and others have found that DIM and its formulated product (BR-DIM manufactured by BioResponse, LLC. with enhanced bioavailability) could down-regulate the expression of AR, Akt and NF-κB, leading to the inhibition of PCa growth and the induction of apoptosis *in vitro* and *in vivo* [21], [22]. DIM was also found to potentiate the therapeutic efficacy of chemotherapeutics [23]. In this study, we investigated whether isoflavone mixture G2535 containing 70.5% genistein, and BR-DIM could inhibit the differentiation of osteoclasts and osteoblasts mediated through regulation of cellular signaling pathways that are involved in bone remodeling and PCa bone metastasis.

## Results

### Differentiation of osteoclasts and osteoblasts in co-culture system with PCa cells

**Figure 1:**
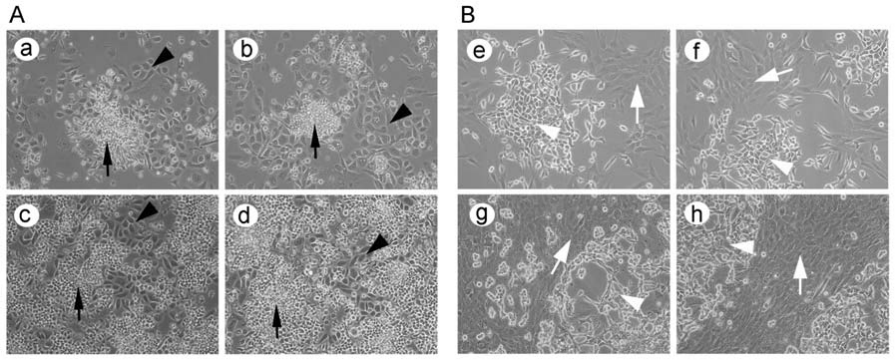
Prostate cancer cell co-culture with osteoclasts or osteoblasts. A. PC-3 cells (indicated by black triangle) were co-cultured with RAW264.7 cells (indicated by black arrow) in low cell density (a, b) and high cell density (c, d). B. C4-2B cells (indicated by white triangle) were co-cultured with hFOB1.19 cells (indicated by white arrow) in low cell density (e, f) and high cell density (g, h). ×100.

**Figure 2:**
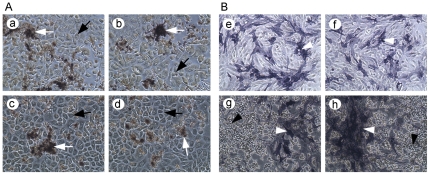
Differentiation of osteoclasts or osteoblasts. A. RAW264.7 cells were co-cultured with PC-3 cells (indicated by black arrow) and treated with 100 ng/ml RANKL (a, b) or 10 ng/ml TGF-β (c, d) for 8 days. TRAP staining showed dark brown granules (indicated by white arrow) in differentiated osteoclasts surrounded by PC-3 cells. B. hFOB1.19 cells were cultured alone (e, f) or co-cultured (g, h) with C4-2B cells (indicated by black triangle) in 39°C for 5 days. Alkaline phosphatase staining showed dark blue granules (indicated by white triangle) in differentiated osteoblasts. ×200.

To investigate the role of bone-related cellular signaling in bone remodeling during PCa bone metastasis, we first developed a co-culture system to allow PCa cells and osteoclasts or osteoblasts grown under the same culture medium condition. We found that PC-3 prostate cancer cells could grow nicely with pre-osteoclast (Raw264.7) cells (Figure 1A) and that both C4-2B cells and pre-osteoblasts (hFOB1.19 cells) could grow together (Figure 1B). Then, we treated co-culture of PC-3 and RAW264.7 cells with RANKL or TGF-β to induce osteoclast differentiation. We incubated the co-culture of C4-2B and hFOB1.19 cells at 39°C to induce osteoblast differentiation. TRAP staining was conducted for detecting osteoclast differentiation while alkaline phosphatase staining was performed for detecting osteoblast differentiation. We observed formation of multinucleated cells and the dark brown granules in the differentiated osteoclasts surrounded by PC-3 cells (Figure 2A), suggesting that the differentiation of osteoclast could be induced by RANKL or TGF-β in PCa growth environment. We also observed dark blue granules in hTOB1.19 cells surrounded by C4-2B cells (Figure 2B), suggesting that osteoblast could be differentiated when grown together with PCa cells.

### TGF-β induced osteoclast differentiation through RANKL

**Figure 3:**
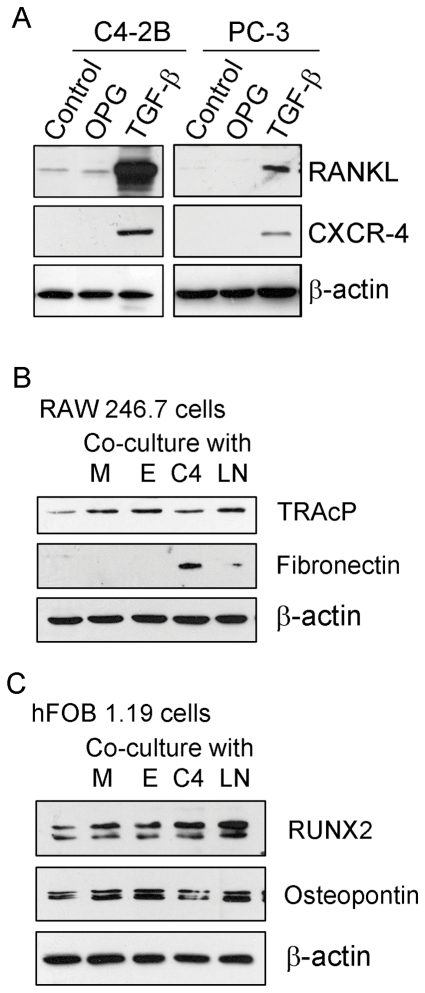
Osteoclast or osteoblast marker analysis. A: C4-2B and PC-3 cells were treated with 100 ng/ml OPG or 10 ng/ml TGF-β for 24 hours. The expression of RANKL and CXCR-4 protein was measured by Western Blot analysis. B and C: RAW246.7 (B) and hFOB1.19 (C) cells in lower chambers were co-cultured with C4-2B (C4), PC-3 (PC), ARCaP_M_ (M) and ARCaP_E_ (E) prostate cancer cells in upper chambers for 48 hours. Western Blot analysis was conducted to measure the expression of osteoclast or osteoblast markers in RAW264.7 or hFOB1.19 cells.

Since we observed differentiation of osteoclast by TGF-β, we tested the effect of TGF-β on the expression of RANKL. We found that the expression level of RANKL was significantly increased by TGF-β treatment in C4-2B and PC-3 cells (Figure 3A), suggesting that the induction of osteoclast differentiation by TGF-β was mediated through the up-regulation of RANKL and that PCa cells could produce RANKL to induce osteoclast differentiation. We also observed that the expression level of CXCR-4, one of the critical genes for metastasis, was significantly increased by TGF-β treatment. These results suggest that the increased level of TGF-β by tumor or stromal cells could promote bone remodeling and PCa metatstasis through RANKL and CXCR-4 signaling.

### Co-culture with prostate cancer cells increased the differentiation of osteoclasts and osteoblasts

To investigate whether PCa cells could promote differentiation of osteoclasts and osteoblasts, we cultured RAW264.7 or hFOB cells in the lower chamber of culture system and cultured various PCa cells including C4-2B, PC-3, ARCaP_M_, and ARCaP_E_ cells in the upper chamber so that the secreted proteins from cancer cells could enter lower chamber from upper chamber through a membrane with 1 µm pore. Western Blot analysis was conducted to measure the osteoclast and osteoblast differentiation markers. We found that co-culture of RAW264.7 cells with PCa cells increased the expression of TRAcP, one of the major osteoclast differentiation markers in RAW264.7 cells (Figure 3B). The expression of fibronectin was also increased when co-cultured with C4-2B and PC-3 cells (Figure 3B). We also observed increased expression of RUNX2, one of the osteoblast markers, in hFOB1.19 cells (Figure 3C). The expression of osteopontin, one of the important genes for bone remodeling, was also induced when co-cultured with PCa cells (Figure 3C). These results suggest that PCa cells could produce some molecules that contribute to the induction of differentiation of osteoclasts and osteoblasts, causing bone remodeling.

### Isoflavone and BR-DIM inhibited the differentiation of osteoclasts and osteoblasts

**Figure 4:**
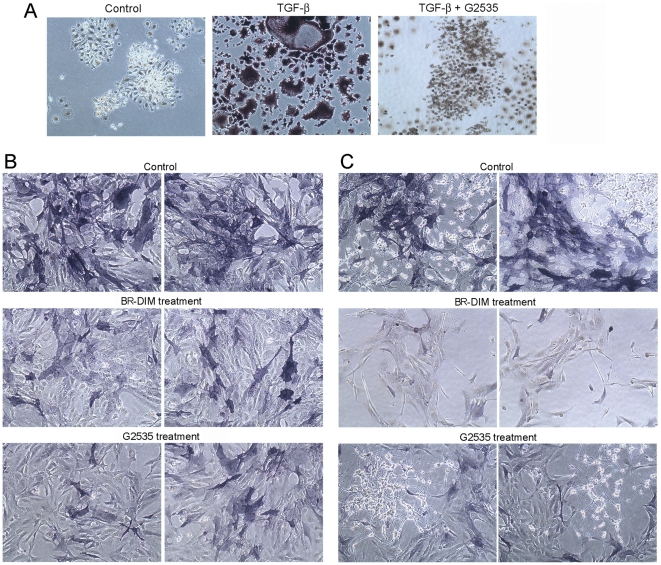
Isoflavone and BR-DIM inhibited the differentiation of osteoclasts and osteoblasts. A: RAW246.7 cells were treated with 10 ng/ml TGF-β alone or combined with 10 µM G2535 for 8 days. TRAP staining was conducted. Dark brown granules indicated the differentiation of osteoclasts. ×100. B and C: hFOB1.19 cells were cultured alone (B) or co-cultured with C4-2B cells (C) at 39°C. The cells were also treated with 20 µM G2535 or 10 µM BR-DIM for 5 days. Alkaline phosphatase staining showed dark blue granules in differentiated osteoblasts. ×200.

To investigate the effects of isoflavone and BR-DIM on the differntiation of osteoclasts and osteoblasts, we treated RAW264.7 or hFOB1.19 cells with 10 to 25 µM G2535 or BR-DIM during the processes of osteoclast and osteoblast differentiation. We conducted TRAP or Alkaline phosphatase staining to detect the differentiation of osteoclasts or osteoblasts. We observed that TGF-β induced formation of multinucleated osteoclasts and that isoflavone and BR-DIM treatments significantly decreased the dark brown granules in RAW264.7 cells (Figure 4A) and the dark blue granules in hFOB1.19 cells when cultured alone (Figure 4B) or co-cultured with C4-2B (Figure 4C). These results clearly suggest that isoflavone and BR-DIM could inhibit the differentiation of osteoclasts and osteoblasts, which could attenuate bone remodeling during PCa metastasis and would be highly desirable for the inhibition of PCa bone metastasis.

### Isoflavone and BR-DIM regulated RANKL and prostate cancer signaling, which could inhibit osteoclastogenesis and prostate cancer growth

**Figure 5:**
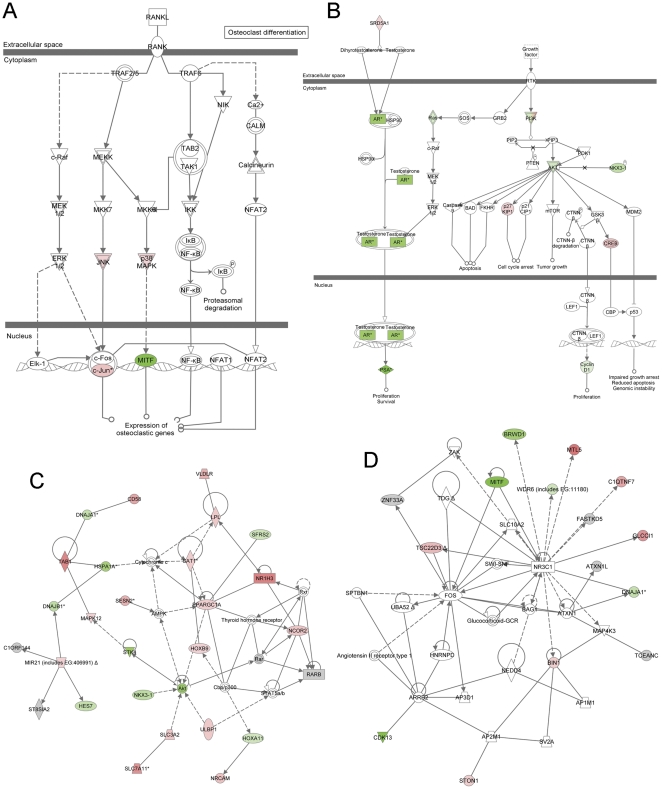
IPA network analysis. BR-DIM regulated the molecules in RANKL signaling (A), prostate cancer signaling (B), and signal transduction networks (C, D) analyzed by microarray and Ingenuity Pathway analysis in C4-2B cells. Red indicates up-regulated molecules while green specifies down-regulated molecules. The figures were automatically created by Ingenuity Pathway Analysis software.

**Figure 6:**
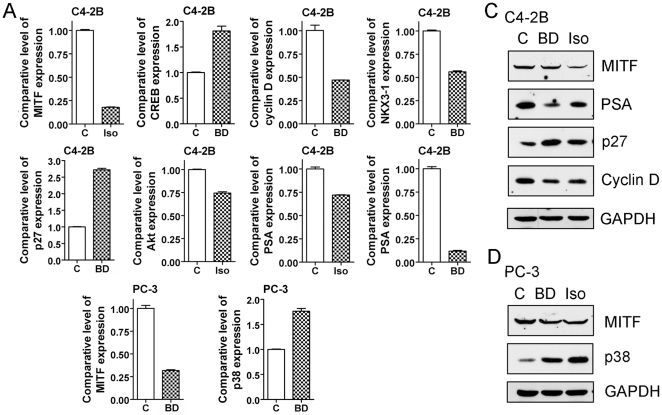
Gene expressions validated by RT-PCR and Western Blot analysis. BR-DIM and isoflavone regulated the expression of MITF, p38, Akt, NKX3-1, p27, cyclin D, CREB and PSA at mRNA and protein levels tested by real-time PCR (A, B) and Western Blot analysis (C, D). C4-2B (A, C) and PC-3 (B, D) cells were treated with 25 µM G2535 or 25 µM BR-DIM for 24 hours (for RNA extraction) or 48 hours (for protein extraction). (BD: BR-DIM).

Since we found that isoflavone and BR-DIM inhibited the differentiation of osteoclast, we further investigated the molecular mechanism of isoflavone and BR-DIM action on osteoclastgenesis. We conducted microarray analysis of gene expression profile of C4-2B cells treated with G2535 or BR-DIM. By using Ingenuity Pathway Analysis, we found that isoflavone and BR-DIM inhibited the cellular signal transduction in the RANKL signaling pathway (Figure 5A and Table 1) and PCa signaling (Figure 5B and Table 1). BR-DIM and isoflavone significantly inhibited the expression of MITF, one of the important transcrption factors involved in the regulation of osteoclastic gene expression (Figure 5A and Table 1). BR-DIM and isoflavone also inhibited the signal transductions in PCa signaling with up-regulation of p27 and down-regulation of PSA and cyclin D (Figure 5B and Table 1). Moreover, isoflavone and BR-DIM regulated the molecules in the signal transduction networks with down-regulation of Akt, NKX3-1, STK-4, CDK13 and MITF (Figure 5C, 5D and Table 1), leading to the inhibition of osteoclastogenesis and PCa growth. Furthermore, we conducted real-time RT-PCR and Western blot analysis to confirm the results from microarray and Ingenity Pathway Analysis. We observed that BR-DIM and isoflavone down-regulated the expression of MITF, Akt, NKX3-1, cyclin D and PSA, and up-regulated the expression of p27, p38 and CREB at the mRNA (Figure 6 A and 6B) and protein (Figure 6C and 6D) levels. These results are consistant with microarray data, suggesting that BR-DIM and isoflavone could inhibit osteoclastogenesis and PCa growth.

**Table 1:** BR-DIM and G2535 altered mRNA expression levels measured by microarray.

Gene	Probe ID	Control	BR-DIM		G2535	
			6 h	48 h	6 h	72 h
PSA	204582_s_at	4219.0	3560.0	11.8	134.0	1388.5
PSA precursor	204583_x_at	5095.8	3424.2	14.6	269.5	1544.8
p27	209112_at	1379.1	1781.6	2823.3	1448.2	2046.0
Cyclin D1	208711_s_at	345.9	182.3	231.9	97.8	110.8
Akt1	207163_s_at	2149.0	926.6	894.4	1223.5	1267.4
NKX3.1	209706_at	1765.4	565.9	259.7	247.0	823.7
MITF	207233_s_at	808.6	735.2	382.4	425.4	526.9
CDK13	207318_s_at	1274.7	910.8	958.6	994.6	1058.7
p38	211561_x_at	728.2	777.7	636.2	813.9	492.8
CREB	214513_s_at	573.8	761.5	588.1	686.7	394.9

### RANKL up-regulated the expression of miR-92a while isoflavone and BR-DIM abragated the up-regulation of miR-92a stimulated by RANKL

**Figure 7:**
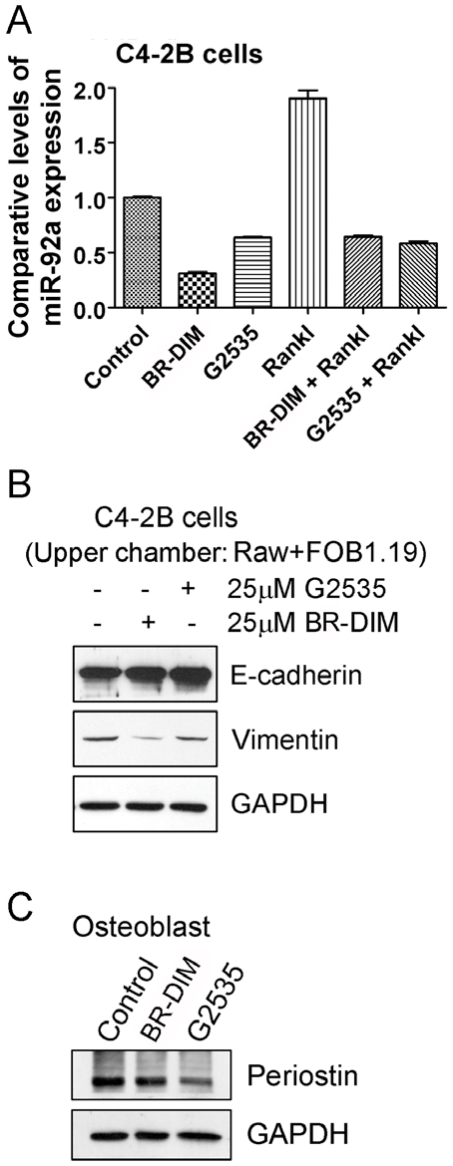
Isoflavone and BR-DIM regulated miRNAs, EMT and osteoblast markers. A: C4-2B cells were treated with 100 ng/ml RANKL, 25 µM G2535, 25 µM BR-DIM, or combination of RANKL and G2535 or BR-DIM for 24 hours. Total RNA was extracted and the expression of miR-92a was detected in treated C4-2B cells. B: C4-2B cells were cultured in lower chamber of co-culture system. RAW246.7 and hFOB1.19 cells were cultured in upper chamber. The cells were treated with 25 µM G2535 or 25 µM BR-DIM for 48 hours. The expression of E-cadherin and vimentin was measured by Western Blot analysis. C: hFOB1.19 cells were treated with 25 µM G2535 or 25 µM BR-DIM for 48 hours. The expression of periostin was detected by Western Blot analysis.

By computerized prediction of target miRNAs, we found that RANKL could regulate several miRNAs such as miR-92a and miR-155, leading to increased osteoclastogenesis. To confirm whether miR-92a is a legitimate target of RANKL or not, we treated C4-2B cells with 100 ng/ml RANKL for 24 hours. We observed that RANKL treatment induced miR-92a expression in PCa cells (Figure 7A). Importantly, we found that treatment of cells with isoflaone or BR-DIM decreased the expression of miR-92a and attenuated the induction of miR-92a expression stimulated by RANKL in PCa cells (Figure 7A), suggesting a mechanistic link between RANKL, miR-92a and the biological activity of isoflavones or BR-DIM.

### Isoflavone and BR-DIM regulated Epithelial-to-Mesenchymal Transition (EMT) markers, E-cadherin and vimentin

Since we observed that isoflavone and BR-DIM could inhibit the expression of miR-92a which could promote metastasis and regulate EMT by targeting down-regulation in the expression of E-cadherin [24], we further investigated the effects of isoflavone and BR-DIM on the expression of E-cadherin and vimentin, the two most important and well accepted EMT markers that are directly associated with EMT phenotype. We cultured C4-2B cells in the lower chamber of a co-culture system where we cultured RAW264.7 and hFOB1.19 cells in the upper chamber to mimic the tumor microenvironment of PCa cells and osteoclast/osteoblast interaction. Subsequently, we treated the cells with 25 µM G2535 or 25 µM BR-DIM for 48 hours. We found that isoflavone or BR-DIM treatment increased the expression of E-cadherin and decreased the expression of vimentin (Figure 7B), suggesting that isoflavone and BR-DIM could prevent the induction of EMT by regulating the expression of miR-92a, E-cadherin and vimentin.

### Isoflavone and BR-DIM inhibited the expression of osteoblastic gene

Since we observed that isoflavone and BR-DIM could inhibit differentiation of osteoblasts, we further investigated the molecular target of isoflavone and BR-DIM action on the osteoblast differentiation. We treated hFOB1.19 cells with 25 µM G2535 or 25 µM BR-DIM for 48 hours. We found that isoflavone or BR-DIM treatment inhibited the expression of periostin (Figure 7C), one of the important genes for osteoblast differentiation. These results suggest that the inhibition of periostin could be one of the molecular mechanisms by which isoflavone and BR-DIM inhibited differentiation of osteoblasts.

## Discussion

It is well known that PCa cells frequently metastasize to the bone. In the bone, metastasized PCa cells could utilize the nutrients from blood in the bone marrow, interact with pre-osteoclasts and pre-osteoblasts, and stimulate bone remodeling [25], [26]. The interaction between PCa cells and pre-osteoclasts/pre-osteoblasts is a critical step for bone remodeling during PCa bone metastasis [26]. Therefore, development of a co-culture system with PCa cells and pre-osteoclasts/pre-osteoblasts is important for investigating molecular interactions of signaling pathways during PCa bone metastasis and bone remodeling. Our data showed that androgen-insensitive PCa cells including PC-3 and C4-2B cells could grow nicely with pre-osteoclasts or pre-osteoblasts in the same culture dish with defined medium, which mimics *in vivo* environment with direct interaction of PCa cells and local bone cells. In our co-culture system, PCa cells, pre-osteoclasts, and pre-osteoblasts could also grow in individual chambers which were separated by a membrane allowing proteins (soluble factors) distribution between the two chambers while separating different types of cells in individual chambers. In this way, the effect of secreted proteins (soluble factors) from PCa cells on bone cells or the effects of secreted proteins from bone cells on PCa cells could be investigated. Using co-culture, we tested the molecular alterations in osteoclasts or osteoblasts stimulated by PCa cells. Recent studies by other investigators showed that co-culture of osteoclast and osteoblast could be useful for examining bone metabolism and osteoclastogenesis [27], [28]. These findings suggest that the co-culture system is very useful for investigating the molecular alterations when PCa cells are homing to the bone, which will allow for assessing alterations in the signal transductions between PCa cells and the local bone cells during PCa bone metastasis and bone remodeling as documented by our results.

**Figure 8:**
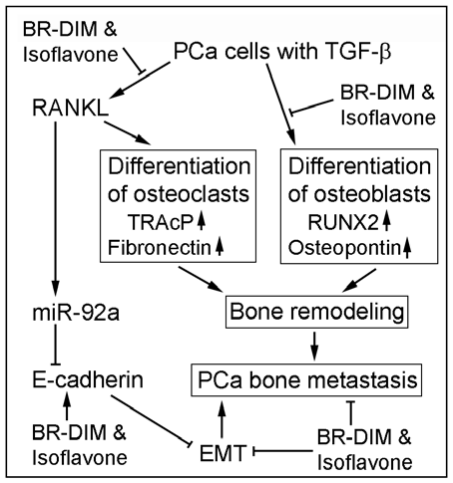
Diagram of isoflavone and BR-DIM regulated bone cell differentiation, bone remodeling, EMT, and PCa bone metastasis.

The differentiation of osteoclasts or osteoblasts is an important step during cancer metastasis to the bone and bone remodeling. By using our co-culture system, we found that pre-osteoclasts could differentiate in to mature osteoclast when co-cultured together with PCa cells. In addition, pre-osteoblasts could also differentiate in to mature osteoblast when co-cultured with PCa cells. Furthermore, we found that co-culture of osteoclasts and osteoblasts with PCa cells could increase the differentiation of osteoclasts and osteoblasts. These findings suggest that the molecules produced by PCa cells could induce the differentiation of osteoclasts and osteoblasts. Importantly, we found that isoflavone and BR-DIM could inhibit the differentiation of osteoclasts and osteoblasts when co-cultured with PCa cells, suggesting that isoflavone and BR-DIM would be useful for the inhibition of PCa bone metastasis and bone remodeling (Figure 8). It has been known that PCa cells in bone metastasis stimulate bone remodeling while bone remodeling facilitates PCa bone metastasis and invasion in the bone [29]–[31], which is known as a vicious cycle of bone remodeling and bone metastasis. Our results showed that isoflavone and BR-DIM inhibited bone cell differentiation, suggesting that isoflavone and BR-DIM could inhibit PCa bone metastasis through disrupting bone remodeling.

It has been reported that differentiation of osteoclasts occurs first during bone remodeling when PCa cells metastasize to the bone [4], [5]. Therefore, inhibition of osteoclast differentiation is important for interrupting PCa bone metastasis and homing. It is well known that RANK/RANKL/OPG signaling is key regulator for osteoclast differentiation [32]. Our results showed that RANKL treatment could significantly induce differentiation of osteoclast, which is consistent with published report [33]. More importantly, we found that TGF-β could up-regulate the expression of RANKL, leading to the differentiation of osteoclasts. These results are also consistent with report from other investigator [31], suggesting that the activation of TGF-β, which is commonly seen in advanced PCa, could stimulate the differentiation of osteoclasts, leading to bone resorption and bone remodeling. Moreover, it is well known that TGF-β could also induce EMT [34], which facilitates PCa progression including invasion and bone metastasis. Importantly, our data showed that isoflavone could attenuate TGF-β induced osteoclast differentiation, and that isoflavone and BR-DIM could regulate the expression of EMT markers (Figure 8), suggesting that these natural agents could be useful for the prevention of PCa progression and bone metastasis.

Emerging evidence suggest that microRNAs (miRNAs) regulate many physiological and pathological processes [35]. Our results showed that in addition to regulating osteoclasts, RANKL could also up-regulate the expression of miR-92a. The miR-92a has been found to down-regulate the expression of anti-tumor genes, resulting in cancer cell proliferation [36]. Recent report showed that E-cadherin is a direct target of miR-92a and that the expression of miR-92a promoted lymph node metastasis of human esophageal squamous cell carcinoma via down-regulation of E-cadherin [24]. Therefore, RANKL could also promote PCa progression via up-regulation of miR-92a and consequently by down-regulation of E-cadherin. Interestingly, isoflavone and BR-DIM were able to attenuate the up-regulation of miR-92a stimulated by RANKL treatment, suggesting the importance of these natural agents (isoflavone and BR-DIM) in the inhibition of PCa progression and bone metastasis (Figure 8).

In addition to the inhibitory effects on osteoclasts, isoflavone and BR-DIM also inhibted the differentiation of osteoblast with down-regulation of periostin, one of the important genes for osteoblast differentiation. Therefore, by targeting both osteoclast and osteoblast differentiation, isoflavone and BR-DIM could effectively interrupt bone remodeling, providing unfavorable micoenvironment for the homing of PCa cells to the bone. Although both isoflavone and BR-DIM showed inhibitory effects on bone cell differentation, the involved molecules and altered levels by isoflavone or BR-DIM treatment were not the same, suggesting that complex regulatory mechanisms are involved. By pathway and network analysis, we found that isoflavone and BR-DIM could affect multiple signaling pathways such as AR/PSA, NKX3-1/Akt/p27, LEF1/cyclin D1, MITF/NR3C1, etc. In BR-DIM treated C4-2B cells, we found more potent down-regulation of PSA which has been found to modulate genes involved in bone remodeling and osteoblast differentiation [37]. These results suggest the multi-targeting effects of isoflavone and BR-DIM, which will make isoflavone and BR-DIM powerful agents for inhibiting PCa growth in bone microenvironment.

In conclusion, isoflavone and BR-DIM were able to target both osteoclast and osteoblast differentiation, and also could target multiple molecules in PCa cells; therefore, these agents could inhibit bone remodeling and PCa growth, suggesting that isoflavone and BR-DIM could be very useful for the prevention of PCa progression, especially bone metastasis. The biological activity of isoflavone and BR-DIM is mediated through the down-regulation of multiple signaling pathways including RANKL, AR/PSA, and Akt signaling, which makes them very promising agents for the prevention and/or treatment of PCa and its bone metastasis in combination with conventional chemotherapy.

## Materials and Methods

### Cell lines, reagents, and antibodies

PC-3 (ATCC, Manassas, VA), C4-2B, ARCaP_E_ and ARCaP_M_ (Novicure, Birmingham, AL) prostate cancer cells and pre-osteoclast RAW264.7 (ATCC) cells were maintained in RPMI 1640 (Invitrogen, Carlsbad, CA) supplemented with 10% fetal bovine serum, 50 U/ml penicillin, and 50 µg/ml streptomycin in a 5% CO_2_ atmosphere at 37°C. Pre-osteoblast hFOB1.19 (ATCC) cells were maintained in DMEM/F12 without phenol red (Invitrogen) supplemented with 10% fetal bovine serum, 50 U/ml penicillin, and 50 µg/ml streptomycin in a 5% CO_2_ atmosphere at 34°C. Isoflavone mixture G2535 (70.5% genistein, 26.3% daidzein and 0.31% glycetein manufactured by Organic Technologies and obtained from NIH) was dissolved in DMSO to make a stock solution containing 50 mM genistein. The concentrations of isoflavone we described in this article all refer to the concentration of genistein in isoflavone mixture. BR-DIM (BioResponse, LLC., Boulder, CO; formulated-DIM with higher bioavailability *in vivo* [38]) was generously provided by Dr. Michael Zeligs and was dissolved in DMSO to make a 50 mM stock solution. Anti-CXCR-4 (Santa Cruz, Santa Cruz, CA), anti-fibronectin (Santa Cruz), anti-RUNX2 (Santa Cruz), anti-osteopontin (Santa Cruz), anti-periostin (Santa Cruz), anti-E-cadherin (Santa Cruz), anti-vimentin (Dako), anti-RANKL (R&D, Danvers, MA), anti-TRAcP (Sigma, St. Louis, MO), anti-MITF (Santa Cruz), anti-p27 (Santa Cruz), anti-PSA (Santa Cruz), anti-cyclin D (Santa Cruz), anti-p38 (Santa Cruz), anti-β-actin (Sigma) and anti-GAPDH (Sigma) primary antibodies were used for Western Blot analysis.

### Co-culture of PCa cells with osteoclasts and osteoblasts

To emulate PCa cell grown in an osteoclastic environment, PC-3 cells and RAW264.7 cells were co-cultured at 1∶1 ratio in RPMI 1640 supplemented with 10% fetal bovine serum, 50 U/ml penicillin, and 50 µg/ml streptomycin in a 5% CO2 atmosphere at 37°C. The cells were treated with 100 ng/ml RANKL or 10 ng/ml TGF-β for 8 days to stimulate RAW264.7 cell differentiation. Likewise, to mimic PCa cell grown in osteoblastic environment, C4-2B and hFOB1.19 cells were co-cultured at 1∶2 ratio in RPMI 1640/F12 (1∶1) supplemented with 10% fetal bovine serum, 50 U/ml penicillin, and 50 µg/ml streptomycin in a 5% CO2 atmosphere at 39°C for 5 days to stimulate osteoblast differentiation of hFOB1.19 cells. Moreover, to investigate the effects of secreted proteins (soluble factors) from one type of cells on to another type of cells, PC-3 or C4-2B PCa cells were cultured in 6 well plate (lower chamber) with RAW264.7 or hFOB1.19 cells grown in cell culture inset (upper chamber, BD Biosciences, Bedford, MA) which has a membrane with 1.0 µm pores opening to lower chamber so that the protein secreted from cells can diffuse to lower chamber while the two types of cells are separated in the lower or upper chambers.

### TRAP staining for testing osteoclast differentiation

RAW264.7 cells or co-culture of RAW264.7 and PC-3 cells were treated with 100 ng/ml RANKL or 10 ng/ml TGF-β for RAW264.7 cells differentiating to osteoclasts. After 8 days of treatment, the formed TRAP in differentiated osteoclasts was stained using TRAP Staining Kit (Sigma) according to the manufacturer's protocol. The purplish to dark brown granules, which indicate the formation of TRAP, were photographed as reported in our earlier publication [39].

### Alkaline staining for testing osteoblast differentiation

hFOB1.19 or co-culture of hFOB1.19 and C4-2B cells were incubated at 39°C for 5 days. The alkaline phosphatase in differentiated osteoblasts was stained using Alkaline Phosphatase Staining Kit (Sigma) according to the manufacturer's protocol. In human bone, alkaline phosphatase activity is restricted to mature osteoblasts. After staining, the blue and dark blue granules, which indicate the presence of alkaline phosphatase, in differentiated osteoblasts were photographed.

### Preparation of total lysates and Western Blot analysis

PC-3, C4-2B, ARCaP_E_, ARCaP_M_, RAW264.7, and hFOB1.19 cells with or without treatments were lysed in RIPA buffer. After centrifugation, the concentration of total protein was measured using BCA protein assay (PIERCE, Rockford, IL). Whole cell lysates were subjected to standard Western Blot analysis as described previously [39]. The signal was then detected using the chemiluminescent detection system (PIERCE, Rockford, IL).

### Microarray analysis of isoflavone and BR-DIM treated PCa cells

C4-2B cells were treated with 25 µM G2535 for 6 and 72 hours or 25 µM BR-DIM for 6 and 48 hours. Total RNA from each sample was isolated by using Trizol (Invitrogen) and purified by using RNeasy Mini Kit and RNase-free DNase Set (QIAGEN, Valencia, CA) according to the manufacture's protocols. cDNA for each sample was synthesized by using Superscript cDNA Synthesis Kit (Invitrogen, Carlsbad, CA). Then, the biotin-labeled cRNA was transcripted *in vitro* (IVT) from cDNA by using BioArray HighYield RNA Transcript Labeling Kit (ENZO Biochem, New York, NY), and purified by RNeasy Mini Kit. The purified cRNA was fragmented and the fragmented labeled cRNA was applied to Human Genome U133 Plus 2.0 Array (Affymetrix, Santa Clara, CA). After washing and staining, the arrays were scanned by using HP GeneArray™ Scanner (Hewlett-Packard, Palo Alto, CA). The gene expressions levels of samples were normalized and analyzed by using Microarray Suite, MicroDB™, and Data Mining Tool software (Affymetrix, Santa Clara, CA). All data is MIAME compliant and the raw data has been deposited in GEO database. The GEO accession number is GSE35324.

### Ingenuity pathway analysis for assessing the effects of isoflavone and BR-DIM on PCa cells

The data from microarray chip were further analyzed for revealing the pathways that are altered by isoflavone or BR-DIM treatment. Signaling pathways altered were analyzed using Ingenuity software (Ingenuity Systems, Redwood City, CA). Potential intracellular signaling pathways or molecule networks affected by BR-DIM treatment were identified using the Ingenuity Pathway Analysis (IPA) software. The significantly altered canonical pathways and predicted molecule networks were automatically created and integrated with microarray results by IPA software.

### Total RNA extraction, miRNA and mRNA detection

Total RNA was extracted by using the miRNeasy Mini Kit and RNase-free DNase Set (QIAGEN, Valencia, CA) following the protocol provided by the manufacturer. Briefly, the cells from each treatment were lysed using QIAzol Lysis reagent. After addition of chloroform, the sample was separated into aqueous and organic phases by centrifugation. The upper aqueous phase was extracted and ethanol was added. The sample was then applied to the RNeasy Mini spin column, where the total RNA including miRNA binds to the membrane. After DNase digestion, DNA, protein, and other contaminants were washed away, and high quality RNA was then eluted in RNase-free water.

The expression level of miR-92a in RANKL, isoflavone and BR-DIM treated C4-2B cells was analyzed by using TaqMan MicroRNA Assay Kit (Applied Biosystems, Foster City, CA) following manufacturer's protocol. Briefly, five nano-gram of total RNA from each sample was subjected to reverse transcription with a specific miRNA primer (Applied Biosystems). Real-time PCR reactions were then carried out in a total volume of 10 µl reaction mixture including RT product, 20× TaqMan miRNA Assay mix, 2× TaqMan PCR Master Mix, and dH_2_O in StepOnePlus (Applied Biosystems). The PCR program was initiated by 10 min at 95°C before 40 thermal cycles, each of 15 s at 95°C and 1 min at 60°C. Data were analyzed according to the comparative Ct method and were normalized by RNU48 and RNU44 expression in each sample.

The expression level of MITF, p27, Akt, cyclin D, NKX3-1, CREB, p38, AR and PSA in isoflavone and BR-DIM treated PCa cells was analyzed by real-time RT-PCR using High Capacity RNA-to-cDNA Kit and SYBR Green Master Mixture from Applied Biosystems. The sequences of primers are shown in Table 2. The PCR was initiated by 10 min at 95°C before 40 thermal cycles, each of 15 s at 95°C and 1 min at 60°C. Data were analyzed according to the comparative Ct method and were normalized by GAPDH expression in each sample.

**Table 2:** The sequences of primers used for real-time PCR.

Gene	Sequence
MITF	F: CCAGGCATGAACACACATTC
	R: TCCATCAAGCCCAAGATTTC
p27	F: CAGGTAGTTTGGGGCAAAAA
	R: ACAGCCCGAAGTGAAAAGAA
Akt	F: TCTATGGCGCTGAGATTGTG
	R: CTTAATGTGCCCGTCCTTGT
Cyclin D	F: GATCAAGTGTGACCCGGACT
	R: TCCTCCTCTTCCTCCTCCTC
NKX3.1	F: CCAGGCTTACTGAGCTGTCC
	R: GCAAAAGGCTCACTCAGTCC
CREB	F: CGACCAGCTGTGTCTGATGT
	R: ATATCCCCTCCCCTTTCCTT
p38	F: TGCACATGCCTACTTTGCTC
	R: AGGTCAGGCTTTTCCACTCA
PSA	F: ATTCCGCCGGAGAGCTGTGTC
	R: TCTCGCACTCCCAGCCTCCC
